# The Viral TRAF Protein (ORF111L) from Infectious Spleen and Kidney Necrosis Virus Interacts with TRADD and Induces Caspase 8-mediated Apoptosis

**DOI:** 10.1371/journal.pone.0037001

**Published:** 2012-05-15

**Authors:** Bai-Liang He, Ji-Min Yuan, Lu-Yun Yang, Jun-Feng Xie, Shao-Ping Weng, Xiao-Qiang Yu, Jian-Guo He

**Affiliations:** 1 MOE Key Laboratory of Aquatic Product Safety/State Key Laboratory of Biocontrol, School of Life Sciences, Sun Yat-sen University, Guangzhou, People's Republic of China; 2 School of Marine Sciences, Sun Yat-sen University, Guangzhou, People's Republic of China; 3 Division of Cell Biology and Biophysics, School of Biological Sciences, University of Missouri-Kansas City, Kansas City, Missouri, United States of America; Karolinska Institutet, Sweden

## Abstract

Infectious spleen and kidney necrosis virus (ISKNV) is the type species of the *Megalocytivirus* genus of the Iridoviridae family. It causes a serious and potentially pandemic disease in wild and cultured fishes. ISKNV infection induces evident apoptosis in mandarin fish (*Siniperca chuatsi*) and zebrafish (*Danio renio*). However, the mechanism is still unknown. After a genome-wide bioinformatics analysis of ISKNV-encoded proteins, the ISKNV open reading frame 111L (ORF111L) shows a high similarity to the tumour necrosis factor receptor-associated factor (TRAF) encoded by fish, mice and mammals, which is essential for apoptotic signal transduction. Moreover, ORF111L was verified to directly interact with the zebrafish TNF receptor type 1 associated death domain protein (TRADD). A recombinant plasmid containing the DNA sequence of ORF111L was constructed and microinjected into zebrafish embryos at the 1–2 cell stage to investigate its biological function *in vivo*. ORF111L overexpression in the embryos resulted in increased apoptosis. ORF111L-induced apoptosis was clearly associated with significant caspase 8 upregulation and activation. The knockdown of zebrafish caspase 8 expression effectively blocked the apoptosis induced by ORF111L overexpression. Significantly, ORF111L overexpression resulted in much stronger effect on caspase 8 and caspase 3 upregulation compared to zebrafish TRAF2. This is the first report of a viral protein similar to TRAF that interacts with TRADD and induces caspase 8-mediated apoptosis, which may provide novel insights into the pathogenesis of ISKNV infection.

## Introduction

The tumour necrosis factor receptor-associated factor (TRAF) family of proteins play essential roles in some signal transduction pathways [1]. Most of mammalian TRAF proteins have an N-terminal RING finger domain and contain a C-terminal TRAF domain, which is composed of a TRAF-N domain (also called coiled-coil) and a highly conserved TRAF-C domain [2]. The N-terminal RING finger domain is important in downstream signalling events [3]. The TRAF-C domain plays an important role in TRAF function by mediating self-association and interactions with other proteins in the apoptosis signalling pathway [3], [4], [5]. For example, mammalian TRAF2 interacts with Tumour necrosis factor Receptor type 1 Associated Death Domain protein (TRADD) and recruits Fas-Associated protein with Death Domain (FADD). The FADD's death domain then combines with cysteinyl aspartate proteinase 8 (caspase 8) and triggers caspase 8-mediated apoptosis [5].

TRAF proteins have been found in 72 species of eukaryotes [6]. TRAF homologues have been found in lower eukaryotes, such as protozoa and unicellular fungi. However, TRAF has not been found in any of the Prokaryota or Archaea species that have been fully sequenced, suggesting that TRAF might have appeared early in the evolution of eukaryotes [6]. Interestingly, only four virus-encoded TRAF-like proteins have been found using protein sequence comparison analyses, namely, turbot reddish body iridovirus (TRBIV) putative TRAF homologue [7], red sea bream iridovirus (RSIV) putative TRAF homologue [8], orange-spotted grouper iridovirus (OSGIV) putative TRAF homologue [9] and infectious spleen and kidney necrosis virus (ISKNV) putative TRAF homologue [6]. TRBIV, RSIV, OSGIV and ISKNV are fish-infecting iridoviruses. Until now, the functions of these iridovirus TRAF-like proteins remain unknown.

ISKNV is the type species of the *Megalocytivirus* genus from the Iridoviridae family. ISKNV causes a serious disease with high mortality rates in mandarin fish (*Siniperca chuatsi*), which severely damages the mandarin fish populations in China. Iridoviruses are icosahedral cytoplasmic DNA viruses that infect invertebrates and poikilothermic vertebrates, including insects, fish, amphibians, and reptiles [10]. Based on the Eighth Report of the International Committee on Taxonomy of Viruses (ICTV), the Iridoviridae family is subdivided into five genera, namely, *Iridovirus*, *Chloriridovirus*, *Ranavirus*, *Lymphocystisvirus*, and *Megalocytivirus*. In recent years, megalocytiviruses have attracted increasing attention because of their ecological and economic effect on wild and cultured fishes. ISKNV and closely related isolates infect a wide range of marine and freshwater fish species, including mandarin fish [11], orange-spotted grouper (*Epinephelus coioides*) [9], large yellow croaker (*Larimichthys crocea*) [12], *Aplocheilichthys normani* [13], turbot (*Scophthalmus maximus*), zebrafish (*Danio rerio*) [14], and more than 50 species of marine fish [15].

As a model system, zebrafish has many advantages compared with mice and other models, including externally fertilized, optically clear embryos because zebrafishes are readily available for observation or manipulation beginning with the 1–2 cell stage embryos [16], [17]. In the ISKNV-infected zebrafish model, viral infection causes tissue necrosis or apoptosis, petechial haemorrhage and kidney and spleen cell enlargement in moribund zebrafish through histological analysis [11], [14]. Furthermore, virus particles are found inside the apoptotic bodies in the apoptotic cells of the spleen tissue under an electron microscope [14]. Moreover, ISKNV infection triggers apoptosis in ISKNV-infected mandarin fish fry cells [14]. Therefore, investigating the role of the viral genes in virus-host interaction is very important, especially in the apoptosis pathway. In the current study, we found that ISKNV ORF111L induces caspase 8-mediated apoptosis in zebrafish model, which might shed light on the pathogenesis of ISKNV infection.

## Materials and Methods

### Zebrafish maintenance

Zebrafish embryos were maintained in Holt buffer with a 14 h light/10 h dark cycle at 28.5°C. Holt buffer is composed of 3.5 g/l NaCl, 0.2 g/l NaHCO_3_, 0.1 g/l CaCl_2_, and 0.05 g/l KCl (PH 7.5) as described in [18]. The embryos stage was defined as hours post-fertilization (hpf) or days post-fertilization (dpf) [19]. Ethical approval for this study was obtained from the ethics committee of the School of Life Sciences, Sun Yat-sen University (Guangzhou, China).

### Collection of ISKNV-infected fish and viral DNA

Moribund mandarin fish (from fish farms in Nanhai, Guangdong Province, China), which showed symptoms of ISKNV infection, were confirmed, collected, and kept at −80°C. ISKNV virus purification was performed as described in [14], [18]. Viral DNA was extracted from the moribund mandarin fishes using a Universal Genomic DNA Extraction Kit Ver.3.0 (TaKaRa, Dalian, China) according to the manufacturer's instructions.

### Bioinformatics analysis

The simple modular architecture research tool (SMART, http://smart.embl-heidelberg.de) was used for the domains analysis in ISKNV ORF111L. Multiple sequence alignments were performed using the ClustalW2 program (http://www.ebi.ac.Uk/clustalw). The results were edited with GeneDoc v 2.6.002 software (http://www.nrbsc.org/gfx/genedoc/index.html) [20]. The phylogenetic tree was constructed using the bootstrap neighbor-joining method of the MEGA4 program [21]. For the phylogenetic tree, 1000 bootstrap analyses were performed. The trees were viewed and outputted with MEGA TreeExplorer [21]. The model structure of the proteins were generated using the SWISS-MODEL Workspace (http://swissmodel.expasy.org/workspace/) [22]. Accession numbers of proteins used in this study are as follow: ISKNV ORF111L, infectious spleen and kidney necrosis virus ORF111L (Accession no. **AAL98835**); TRBIV-PTH, turbot reddish body iridovirus predicted TRAF homology (Accession no. **ADE34445.1**); RSIV-PTH, red sea bream iridovirus predicted TRAF homology (Accession no. **BAD98248**); OSGIV-PTH, orange-spotted grouper iridovirus predicted TRAF homology (Accession no. **AAX82417**); Hs-TRAF, *Homo sapiens* TRAF (Accession no. **CAI15105**); Ms-TRAF, *Mus musculus* TRAF (Accession no. **AAC37662**); Rat-TRAF, *Rattus norvegicus* TRAF (Accession no. **AAI69064.1**); Dr-TRAF1, *Danio rerio* TRAF1 (Accession no. **NP_001121853**); Dr-TRAF2, *Danio rerio* TRAF2 (Accession no. **CAM15136**); Dr-TRAF3, *Danio rerio* TRAF3 (Accession no. **AAH77157**); Dr-TRAF4, *Danio rerio* TRAF4 (Accession no. **CAD89006**); Dr-TRAF5, *Danio rerio* TRAF5 (Accession no. **XP_692341**); Dr-TRAF6, *Danio rerio* TRAF6 (Accession no. **Q6IWL4**); Dr-TRAF7, *Danio rerio* TRAF7 (Accession no. **NP_001073654**); Tg-TRAF, *Taeniopygia guttata* TRAF (Accession no. **XP_002190587.1**); Gg-TRAF, *Gallus gallus* TRAF (Accession no. **XP_415560.2**); Ec-TRAF, *Equus caballus* TRAF (Accession no. **XP_001497958.2**); Cf-TRAF, *Canis familiaris* TRAF (Accession no. **XP_537792.2**); Xenopus-TRAF, *Xenopus (Silurana) tropicalis* TRAF (Accession no. **XP_002942767.1**); Of-TRAF, *Oplegnathus fasciatus* TRAF (Accession no. **ACV04846.1**); Ci-TRAF, *Ctenopharyngodon idella* TRAF (Accession no. **ABE99696.1**); Tn-TRAF, *Tetraodon nigroviridis* TRAF (Accession no. **CAG05243.1**); Om-TRAF, *Oncorhynchus mykiss* TRAF (Accession no. **CAD69021.2**) and Bb-TRAF, *Branchiostoma belcheri* TRAF (Accession no. **ABN04151.1**).

### GST pull-down assay

To express the GST-111L fusion proteins, two primers (Table 1) were used to amplify the full length of *ISKNV ORF111L* from the ISKNV genomic DNA. The fragments were cloned into the pGEX-4T-1 vector (GE Healthcare Life Sciences, USA). This GST-111L-expressing plasmid was designated as pGST-111L.

**Table 1:** Summary of primers used in this study.

Primers	Primer sequence (5′-3′)
**Overexpression assay**	
111L-GFP-F	CGGAATTCATGGAACTGTGTCAGCCC
111L-GFP-R	CGGGATCCGAGATCGCACACGTGTA
RFP-111L-F	GGGGTACCATGGAACTGTGTCAGCC
RFP-111L-R	CGGGATCCGAGATCGCACACGTG
111-RNA-F	ATGGACTACAAAGACGATGACGACAAGATGGAACTGTGTCAGCCCAACAAC
111-RNA-R	CTAGAGATCGCACACGTGTACCTTGAT
**GST Pulldown assay**	
GST-111L-F	CGGGATCCATGGAACTGTGTCAGCCC
GST-111L-R	CGGAATTCGAGATCGCACACGTGTA
MYC-TRADD-F	GGGGTACCAATGGACAGTATAGACACAAAGAGG
MYC-TRADD-R	CGGGATCCTTAATCTCGTGGCTGGAT
**RT-qPCR assay**	
β-actin-QF	ATGCCCCTCGTGCTGTTTTC
β-actin-QR	GCCTCATCTCCCACATAG GA
caspase 8-QF	AGACCAGGAACAAGGAGGCAGACT
caspase 8-QR	CTGTAGTAATTGTGCCAGCCGAAGAG
**ISH riboprobe synthesis**	
caspase 8-PF	CGGAATTCCTGACAAGCGGTGATGTGGACC
caspase 8-PR	GGGGTACCCATATCAGTGCCTGTTCGTTTGAGC
caspase 3-PF	ATGCAGGTTGATGCCAAGCCT
caspase 3-PR	TGAACAGACTAGTTAAAGACTTGAGATCCAC
**Morpholino assay**	
MO-caspase 8-F	CTGACAAGCGGTGATGTGGA
MO-caspase 8-R	GCCCAAGCCTCTGTTGTTTT
MO-β-actin-F	GACGACCCAGACATCAGGGAGTG
MO-β-actin-R	TGGAGTTGAAGGTGGTCTCGTGGA

The underlined letters is the restriction endonuclease cutting site.

To express the MYC-TRADD fusion proteins, the total RNA was extracted from adult zebrafish using an SV Total RNA Isolation (Promega, USA). The cDNA was then synthesized with MMLV (Promega, USA). Two primers (Table 1) were used to amplify the full length *TRADD* from zebrafish cDNA, and the fragments were cloned into the pMYC-CMV vector (Clontech, Takara Bio Company, Japan). The resulting MYC-TRADD-expressing plasmid was designated as pMYC-TRADD.

Human embryonic kidney 293T (HEK293T) cells were cultured in Dulbecco's Modified Eagle's Medium with 10% FBS in 5% CO_2_. The pMYC-TRADD plasmid was transfected into the 293T cells in 10 cm plates using Lipofectamine 2000™ (Invitrogen, USA) according to the manufacturer's instructions. At one day post-transfection, the 293T cells were washed with cold PBS and lysed with RIPA buffer (Sigma, USA). The supernatant liquids were then collected by centrifugation at 16,000×g for 10 min at 4°C. At the same time, the GST-111L fusion protein or GST protein from *Escherichia coli* cells were harvested and added into the beads. Subsequently, the 293T cell supernatant liquids (containing the MYC-TRADD fusion proteins) were added into the GST proteins or GST-111L fusion proteins binding beads. A GST pull-down assay was then carried out according to the manufacturer's instructions (MagneGST™ Pull-Down System, Promega, USA). Finally, the captured MYC-TRADD fusion proteins were separated using 1× SDS loading buffer, and was detected through western blot analysis using an anti-MYC antibodies (Invitrogen, USA).

### Plasmid construction and microinjection into the zebrafish embryo

Two primers (Table 1) were used to amplify the full length of *ISKNV ORF111L* from the ISKNV genomic DNA. The fragments were digested and cloned into the pEGFP-N3 or pdsRed2-C1 vector (Takara Bio Company, Clontech, Japan). This 111L-EGFP and RFP-111L-expressing plasmid was designated as p111L-GFP and pRFP-111L, respectively.

The plasmids were linearised and purified using a QIAquick PCR Purification Kit (Qiagen, USA), and then resuspended in water at 150 ng/µl. The linearised plasmid was microinjected into 1–2 cell stage zebrafish embryos using an IM 300 Microinjector (Narishige, JAPAN) at 1 nl per embryo. On the other hand, full length of *ISKNV ORF111L* was PCR amplified and cloned into the pGEM-T-easy vector (Promega, USA). Then the capped and poly (A) tailed ORF111L RNA was synthesized *in vitro* according to the manufacturer's instructions (Ambion's mMESSAGE mMACHINE and Poly (A) Tailing Kit, USA). The synthesized RNA was microinjected into 1–2 cell stage embryos to overexpress ISKNV ORF111L (200 pg/embryo). The embryonic development of zebrafish was visualized and recorded using an OlympusDP71 digital camera mounted onto an OLYMPUS MVX10 fluorescence stereomicroscope.

### Hematoxylin-eosin (HE) staining

Hematoxylin has a deep blue-purple colour and stains nucleic acids by a complex, incompletely understood reaction. Eosin is pink and stains proteins nonspecifically. In a typical tissue, nuclei are stained blue, whereas the cytoplasm and extracellular matrix have varying degrees of pink staining [23]. For hematoxylin-eosin (HE) staining, embryos samples were collected and treated as described [24]. Specimens were sectioned at 5 µm using a Leica RM2145 microtome. HE staining was subsequently performed using standard protocols [25].

### Terminal deoxynucleotidyl transferase-mediated fluorescein-dUTP nick end labelling (TUNEL) assay

TUNEL has become one of the main methods for detecting apoptosis [26]. Injected embryos were collected and fixed overnight in 4% paraformaldehyde (PFA, Sigma, USA) at 4°C. After washing with PBST, the embryos were dechorionated, dehydrated into 100% methanol and maintained at −20°C. Prior to staining, the embryos were rehydrated in PBST, post-fixed in 4% PFA for 1 h, blocked in TdT buffer, and incubated with a mixture of TdT enzyme solution and fluorescein-labelled dUTP (Roche Applied Science) for 60 min at 37°C. The samples were analyzed in a drop of PBS under a fluorescence microscope. For staining, the embryos were then washed twice with PBS and exposed for 30 min to anti-fluorescein antibodies conjugated with alkaline phosphatase (Roche Applied Science) at 37°C. The signal was detected using NBT/BCIP staining, and images were captured.

### Real-time quantitative PCR (RT-qPCR) analysis

Thirty RFP-111L-overexpressing embryos were collected at 1, 2, 3, 4, and 5 dpf. Thirty wild-type and empty vector-injected embryos were used as controls and collected at the same time points. Total embryonic RNA was isolated and then reverse transcribed into cDNA as previously described [18]. Two caspase 8 specific primers (Table 1) were used to analyze the caspase 8 transcription quantitatively in the embryos at different time points. The caspase 8 transcription was assayed in triplicate for each time point and zebrafish β-actin was used to normalize the starting RNA quantity. All amplifications and detection were carried out in a LightCycler480 System (Roche, Germany) as previously described [14]. The values were normalized to the corresponding β-actin values to determine the relative copy number. The RNA quantification levels were calculated using the 2^−ΔΔCt^ relative quantification method [27], [28]. The relative copy number was then used to calculate the fold change of the caspase 8 transcription at different time points.

### Caspase 8 activity assay

Caspase 8 activity was test by caspase 8-Glo assay (Promega, Madison, WI, USA). The caspase 8-Glo assay provides a luminogenic caspase 8 substrate in a buffer system. Zebrafish embryos were microinjected with pRL-TK (express low to moderate levels of Renilla luciferase as an inner control, Promega, USA), pRL-TK+pdsRed2-C1 or pRL-TK+pRed-1111L. At 1 dpf, 2 dpf, 3 dpf, 4 dpf, and 5 dpf, 20 embryos were collected and lysed as previously described [29]. After adding the cell lysate to the caspase 8-Glo substrate, the substrate was cleavage by cellular caspase 8 and a luminescent signal (produced by the firefly luciferase reaction) was generated. The Firefly and Renilla luciferase activity was measured by a TECAN infinite 200 luminometer according to the manufacturer's instructions. After normalization to Renilla luciferase, the relative luciferase (caspase 8) activity was calculated.

### Morpholino-mediated gene knockdown assay

Recently, morpholino (MO, Gene Tools, LLC, USA)-mediated gene knockdown has been successfully used in zebrafish embryo [30], [31], [32]. An organism that has been treated with MO to temporarily knockdown the expression of a targeted gene is called a morphant. The sequence of caspase 8 splice-blocking MO (caspase 8^Spl-MO^) was 5′-ACAGGGTTTTAACTCACAGTAGATC-3′, and interferes with the splicing junction at exon 3/intron 3, which results in a truncated protein [33]. The caspase 8^Spl-MO^ solution was mixed with the linearised plasmid pdsRed2-C1 or pRFP-111L before injection into the 1–2 cell stage embryos. The working concentrations of the MO and plasmids were 1.5 mM and 150 ng/µl, respectively.

### Whole mount RNA in situ hybridization (WISH) assay

The specific mRNA transcription was measured and localized in the whole mount zebrafish embryos using digoxigenin-labelled complementary RNA probes. The partial cDNA (634 base pairs length) sequence of caspase 8 and caspase 3 was PCR amplified, and the fragments were cloned into a pGEM-T-easy vector (Promega, USA) to generate an antisense digoxigenin-labelled RNA probe (DIG RNA Labelling Kit, Roche Applied Science, Germany) *in vitro* for the WISH assay in zebrafish embryo.

Embryos were fixed overnight in 4% PFA at 4°C. The samples collected after 24 hpf were incubated in 0.003% 1-phenyl-2-thiourea (PTU, Sigma, USA) to remove any pigments prior to fixation [34], [35]. Before probing, the embryos were rehydrated and, if older than 24 hpf, treated with proteinase K (10 µg/ml) at room temperature for 5 min to 50 min, depending on the development stage. The embryos were then refixed in 4% PFA for 1 h, and prehybridized in 57% formamide hybridization buffer for 4 h at 65°C. The buffer was replaced with 57% formamide hybridization buffer containing 1 ng/µl of the digoxigenin-labelled probe, and hybridization was carried out overnight at 65°C. After blocking, the embryos were exposed overnight to sheep anti-digoxigenin Fab antibodies conjugated with alkaline phosphatase (Roche Applied Science, Germany) at 4°C. The signal was detected using NBT/BCIP staining and images were captured.

### Statistical analysis

Comparisons between groups of numerical data were evaluated using paired Student's *t* tests. Data are expressed as means ± standard error of the mean (SEM). *P*-values<0.05 or <0.01 were considered statistically significant, and is represented with an asterisk (*) or (**), respectively. All experiments were repeated at least three times.

## Results

### Bioinformatics analysis of *ISKNV ORF111L*

**Figure 1:**
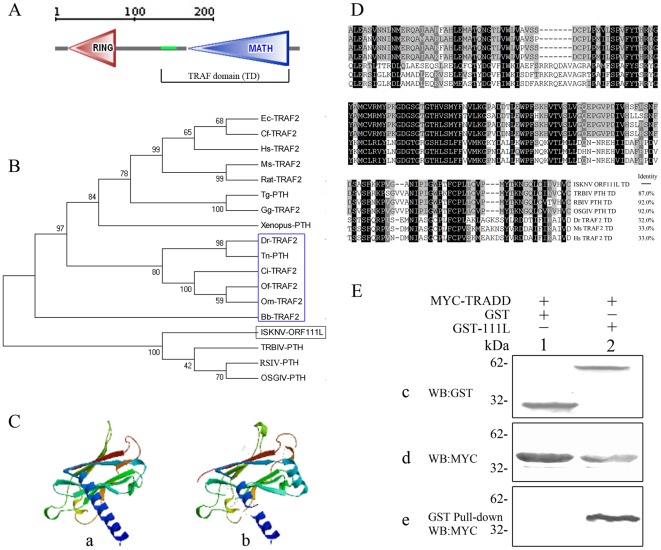
Bioinformatics analysis and protein interaction of ISKNV ORF111L. (A) ISKNV ORF111L domain architecture was predicted by SMART program. (B) Phylogenetic tree of ISKNV ORF111L with other TRAF family proteins. The Bootstrap test of phylogeny was calculated 1000 replicates. The numbers at the nodes indicate bootstrap values. (C) Homology model structures analysis of TRAF domain from ISKNV ORF111L (panel a) and *Homo sapiens* (panel b). (D) Multiple sequence alignments of TRAF domain from different TRAF proteins. PTH, predicted TRAF homology; TD, TRAF domain. (E) Expression of GST proteins (c, lane 1), GST-111L fusion proteins (c, lane 2) and MYC-TRADD fusion proteins (d, lanes 1 and 2) were effective. After the GST pull down assay, MYC-TRADD fusion proteins were detected in the GST-111L sample (e, lane 2) but not in the GST control sample (e, lane 1), indicating the interaction between ISKNV ORF111L and zebrafish TRADD.

*ISKNV ORF111L* is 891 base pairs (bp) long and is predicted to encode a 296- amino acid (aa) protein. ISKNV ORF111L contains an N-terminal zinc finger RING domain and a C-terminal TRAF domain (TD) composed of a coiled-coil domain and a MATH (meprin and TRAF homology) domain (Fig. 1A).

To explore further the evolutionary origin of TRAF isoforms, diverse TRAF homology sequences were searched in the NCBI and Ensembl databases and a phylogenetic tree analysis was performed. Representative TRAF homology sequences were chosen from *Xenopus*, chicken, dog, house, mouse, rat, human, four megalocytiviruses (ISKNV, TRBIV, RSIV, and OSGIV), and six fish species. The result shows that the ISKNV ORF111L and the TRAF homologues from TRBIV, RSIV, and OSGIV belong to one monophyletic group. Evolutionarily speaking, this group is closest to fish (Fig. 1B, blue box) but relatively distant from the TRAFs of *Xenopu*s, chicken, and mammals (Fig. 1B).

The TRAF domain from the TRAF family of proteins plays an important role in the upstream and downstream interactions, and is essential in the apoptosis pathway [3], [4], [5], [36], [37], [38]. The model structures of the TRAF domain were generated using the SWISS-MODEL Workspace. The TRAF domain of *ISKNV ORF111L* (Fig. 1C, panel a) shows high similarity with that of *Homo sapiens* (Fig. 1C, panel b). The TRAF domain of ISKNV ORF111L shows high similarity with those of virus and vertebrate species, and has 87.0% identity with turbot reddish body iridovirus, 92.0% identity with both the red sea bream iridovirus and the orange-spotted grouper iridovirus, 32.0% identity with *Danio rerio*, and 33.0% identity with both *Mus musculus* and *Homo sapiens* (Fig. 1D).

### ISKNV ORF111L directly interacted with zebrafish TRADD

TRAF proteins were involved in several signalling pathways, including apoptosis, which plays an important role in cell fate and viral infections [39]. A GST pull-down assay was performed to investigate the interaction between ISKNV ORF111L and zebrafish TRADD. The expression of the GST control proteins (Fig. 1E, panel c, lane 1, 27 kDa), GST-111L fusion proteins (Fig. 1E, panel c, lane 2, 60 kDa) from bacteria lysate, and the MYC–TRADD fusion proteins from transfected cells (Fig. 1E, panel d, lanes 1 and 2, 35 kDa) were confirmed effective through western blot analysis using anti-GST and anti-MYC antibodies, respectively. The GST pull-down assay was performed according to the manufacturer's instructions. MYC–TRADD fusion proteins were detected in the GST-111L sample (Fig. 1E, panel e, lane 2, 35 kDa). However, no band corresponding to MYC–TRADD was detected in the GST control sample (Fig. 1E, panel e, lane 1), which clearly indicates the interaction between ISKNV ORF111L and zebrafish TRADD.

### ISKNV ORF111L overexpression resulted in abnormal phenotype in zebrafish embryo

**Figure 2:**
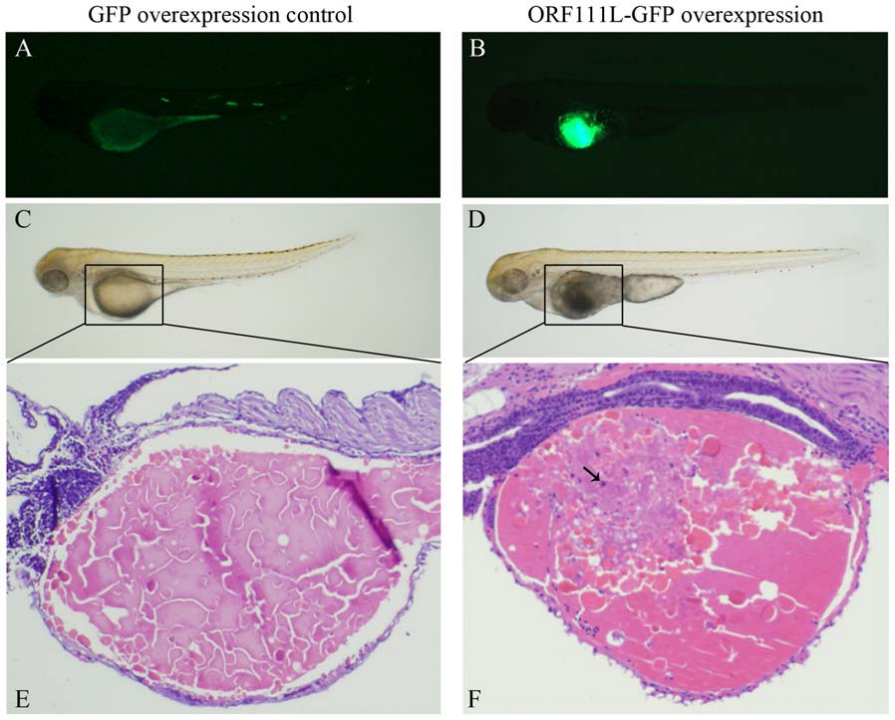
ISKNV ORF111L overexpression resulted in abnormal phenotype in zebrafish embryo. (A–B) The GFP and 111L-GFP expression in pEGFP-N3- and p111L-GFP-injected embryos were shown. (C–D) Empty vector pEGFP-N3-injected embryo did not show any phenotypic abnormality, while the yolk sac was not completely developed in 111L-GFP overexpressing embryo. (E–F) Hematoxylin-eosin staining assay was performed in GFP- and 111L-GFP-overexpressing embryo.

To elucidate the functions of ISKNV ORF111L *in vivo*, the full length of *ISKNV ORF111L* was cloned into the GFP-expressing vector pEGFP-N3. This recombinant plasmid was microinjected into 1–2 cell stage embryos to overexpress 111L-GFP. The pEGFP-N3-injected embryo, which developed healthily, was used as the control (Fig. 2A and 2C). 111L-GFP overexpression resulted in a severely abnormal phenotype in the zebrafish embryos. The 111L-GFP-overexpressing tissues, especially the yolk sac, did not develop healthily (Fig. 2B and 2D). Hematoxylin-eosin staining showed that numerous nucleus staining cells (deep blue-purple colour) were observed in 111L-GFP-overexpressing yolk sac tissues (Fig. 2F), and it was not found in empty vector-injected control embryo (Fig. 2E).

### ISKNV ORF111L-overexpressing embryo showed evident apoptosis

**Figure 3:**
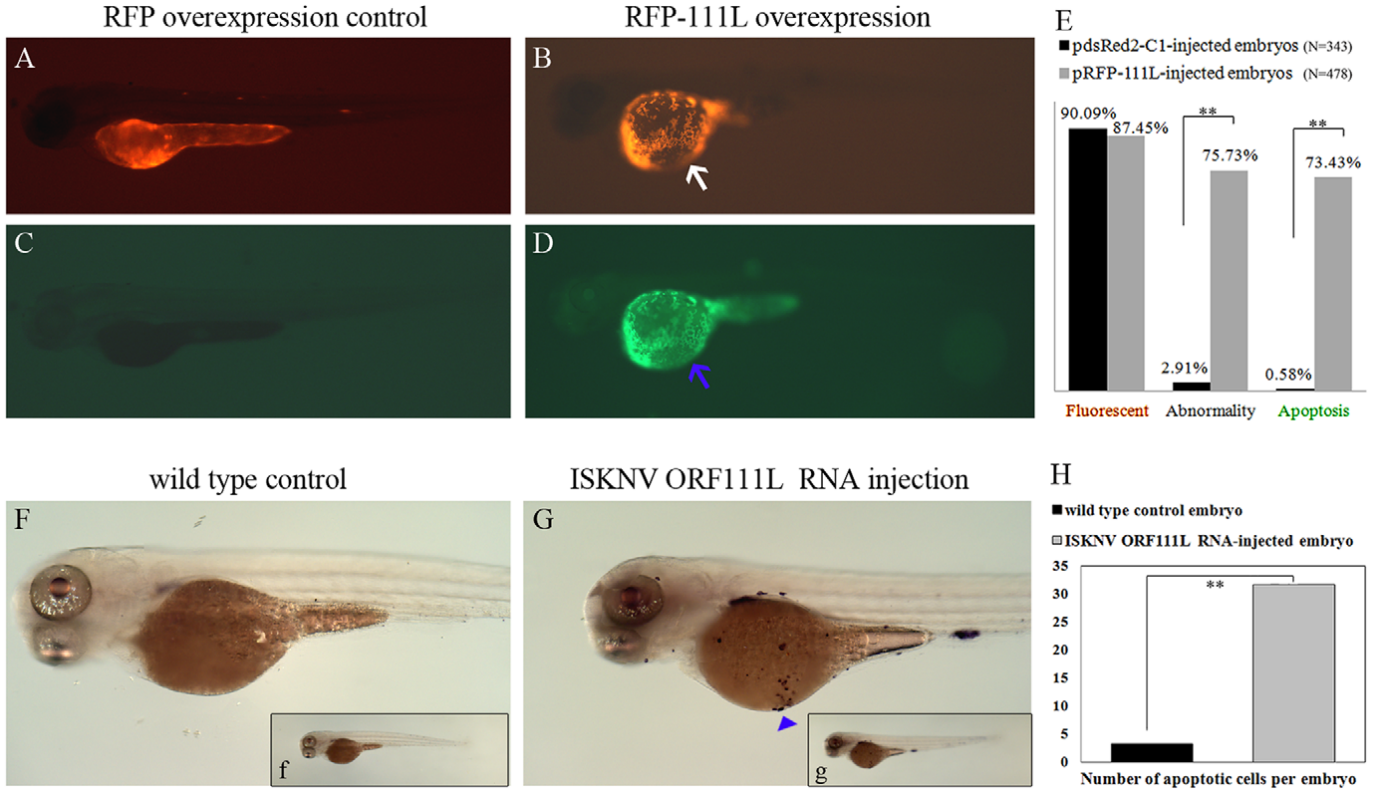
ISKNV ORF111L overexpression resulted in evident apoptosis in zebrafish embryo. (A–B) The RFP and RFP-111L expression in pdsRed2-C1- and pRFP-111L-injected embryos were shown. (C–D) No obvious apoptotic signal was found in RFP overexpressing embryo, while increased apoptosis signal were clearly observed in RFP-111L-overexpressing embryo. (E) Statistics analysis of fluorescent, abnormality and apoptosis of embryos. Embryos shown above were at 3 dpf stage and represented the typical phenotype in three individual microinjection experiments. The significance of differences are calculated by the *t*-test (** indicates *p*<0.01). (F–G) TUNEL assay was performed in wild type and ISKNV ORF111L mRNA-injected embryos. Apoptotic cells were shown (arrow head). Figure F and G is the enlarged figure from panel f and g, respectively. (H) After the NBT/BCIP staining, the number of apoptotic cells was counted in wild type and ISKNV ORF111L mRNA-injected embryos.

To demonstrate that the ORF111L-induced abnormal phenotype was not GFP reporter gene-dependent, the full length of *ISKNV ORF111L* was cloned into the RFP-expressing vector pdsRed2-C1. RFP-111L overexpression resulted in similar abnormality compared with that of 111L-GFP. Statistically, 75.73% of the pRFP-111L-injected embryos and 2.91% of the pdsRed2-C1-injected control embryos were abnormal at 3 dpf (Fig. 3E), which indicates that the abnormal phenotype was induced by ISKNV ORF111L overexpression but not by physical trauma. To investigate whether ISKNV ORF111L-induced abnormal phenotype is associated with apoptosis, a TUNEL assay was performed on RFP- or RFP-111L-overexpressing embryos. 73.43% of ISKNV ORF111L-overexpressing embryos showed increasing positive cell death signal compared with only the 0.58% in those of pdsRed2-C1-injected control embryos (Fig. 3E). Though the emission spectra of fluorescein (apoptotic signal) is relative close to RFP, there is no apoptotic signal in RFP-overexpressing embryo (Fig. 3C) in our experimental procedure, indicating that 111L-RFP overexpression-induced apoptosis is reliable (Fig. 3D, arrow).

Moreover, we directly overexpressed ORF111L by microinjecting ISKNV ORF111L mRNA (in vitro synthesis, capped and poly A tailed) into 1–2 cell stage embryos. TUNEL assay was performed by incubating the ORF111L mRNA-injected embryos with a mixture of TdT enzyme solution and fluorescein-labelled dUTP Then the embryos were subsequently incubated with an alkaline phosphatise conjugated anti-fluorescein antibody. After NBT/BCIP staining, apoptotic cells were clearly observed in ISKNV ORF111L mRNA-injecting embryos (Fig. 3G and 3g, arrow head), but not in the wild type control embryos (Fig. 3F, 3f and 3H). This finding suggests that the ISKNV ORF111L-induced abnormal phenotype in zebrafish embryos is associated with evident apoptosis.

### ISKNV ORF111L overexpression significantly activated zebrafish caspase 8

**Figure 4:**
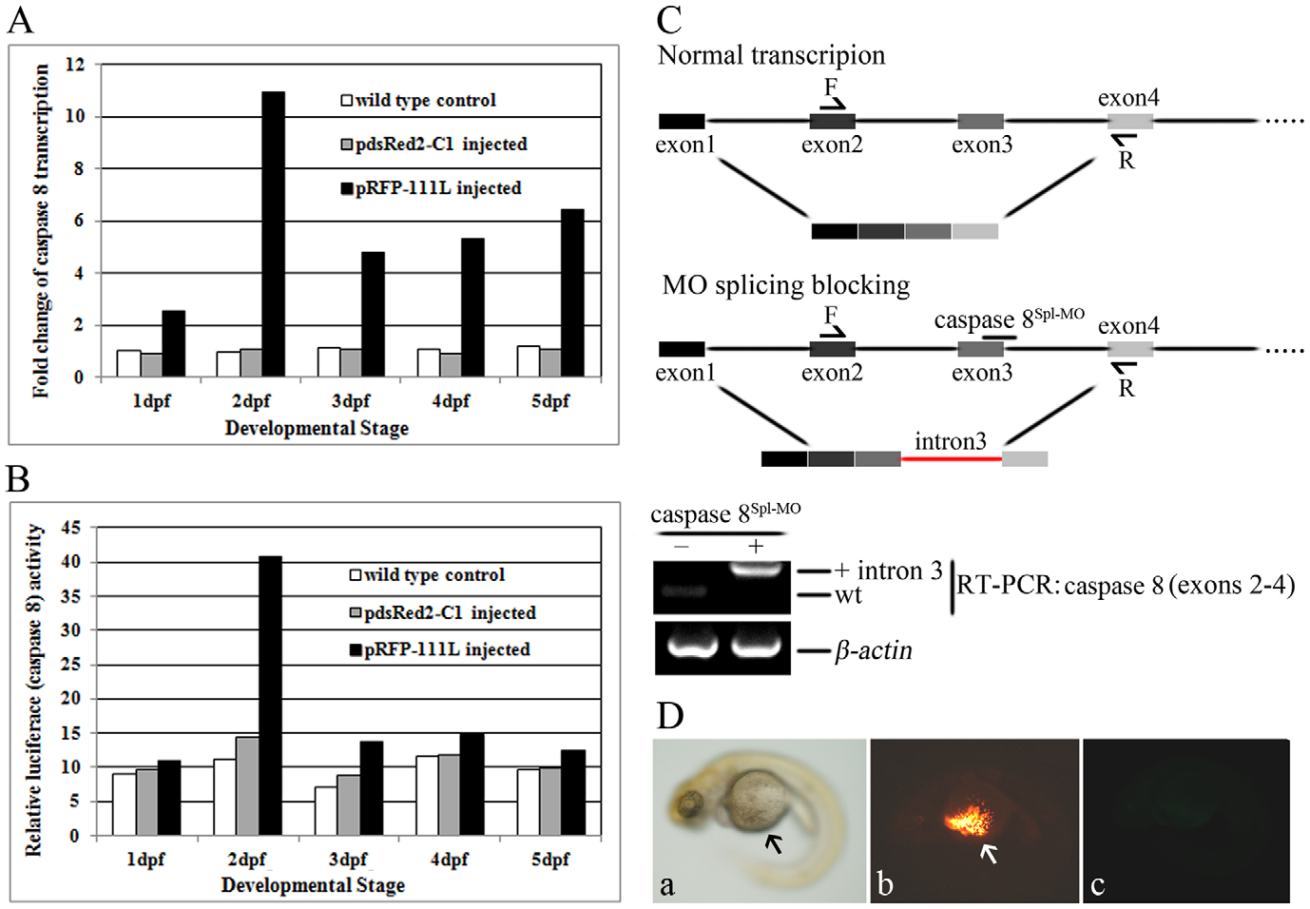
ISKNV ORF111L-induced apoptosis was mediated by caspase 8. (A) The caspase 8 expression was tested by RT-qPCR assay. The expression level of *β-actin* was set as 1, and values were normalized to the corresponding *β-actin* values to determine the relative copy number. All data are presented as means from three individual injection experiments. (B) The caspase 8 activity was tested in wild type, pdsRed2-C1- and pRFP-111L-injected embryos. (C) Knockdown efficiency of caspase 8^Spl-MO^ in zebrafish embryos. Schematic representation of normal transcription of caspase 8 and morpholino splicing blocking of caspase 8 was shown. The caspase 8^Spl-MO^ interferes with splicing junction at exon 3/intron 3, resulting in retention of the intron 3. The retention of intron 3 resulted in a frame-shift and the truncation of protein translation. Knockdown effect of caspase 8^Spl-MO^ was tested in by RT-PCR. Forward primer is located in exon 2, and the reverse primer is located in exon 4. Result showed that caspase 8^Spl-MO^ strongly depletes the wild type caspase 8 mRNA and had a high gene knockdown effect. (D) Caspase 8 knockdown effectively blocked ISKNV ORF111L-induced apoptosis. The caspase 8^Spl-MO^ and pRFP-111L were co-injected into 1–2 cell stage embryos. The yolk sac of embryo was healthily developed (panel a). The expression of RFP-111L fusion proteins were clear (panel b), and no obvious apoptosis signal were found in TUNEL assay (panel c), indicating that knockdown the expression of caspase 8 could effectively block ISKNV ORF111L-induced apoptosis. Embryos shown above are the typical phenotype in three independent experiments.

Iridovirus-induced apoptosis has been associated with caspase activation [40], [41], [42], [43]. To investigate the ISKNV ORF111L-induced apoptotic signal pathway, caspase 8 expression was detected in the ISKNV ORF111L-overexpressing embryos using RT-qPCR. As shown in Fig. 4A, the caspase 8 expression in the pRFP-111L-injected embryos was significantly upregulated by 2.5-,10.9-, 4.7-, 5.3-, and 6.4-fold compared with the wild-type or pdsRed2-C1-injected embryos at 1, 2, 3, 4, and 5 dpf, respectively. Moreover, the caspase 8 activity was also upregulated by ORF111L overexpression (Fig. 4B), which consolidated the caspase 8 upregulation by RT-qPCR assay.

### Caspase 8 knockdown effectively blocked ISKNV ORF111L-induced apoptosis

To characterize the ISKNV ORF111L-induced apoptotic pathway molecularly, we capitalized on the unique advantages of zebrafish embryos for *in vivo* gene knockdown. Specifically, zebrafish caspase 8 was blocked down to determine the effects of ISKNV ORF111L overexpression on the embryos.

The gene knockdown efficiency of caspase 8 was first tested by using a splicing site targeting morpholino (caspase 8^Spl-MO^) in zebrafish embryos. The caspase 8^Spl-MO^ interfered with the splicing at the exon3/intron3 splice junction, and resulted in intron3 retention (Fig. 4C). This aberrant splicing product resulted in an early stop codon (as result of the in-frame stop reading of intron 3), and was predicted to remove a part of the second DED domain and the entire catalytic domain of zebrafish caspase 8. The reverse transcription PCR shows that caspase 8^Spl-MO^ effectively depleted the WT caspase 8 mRNA pool (Fig. 4C).

The effects of caspase 8^Spl-MO^ on ISKNV ORF111L-overexpressing embryos were then tested. The plasmid pRFP-111L was co-injected with caspase 8^Spl-MO^ into 1–2 cell stage zebrafish embryos. The co-injected embryo revealed no yolk sac abnormality (Fig. 4D, panel a, black arrow), the RFP-111L expression was clear (Fig. 4D, panel b, white arrow), and no obvious apoptotic signal was found (Fig. 4D, panel c). These findings clearly indicate that ISKNV ORF111L-induced apoptosis is mediated by caspase 8

### Comparison between ISKNV ORF111L and its cellular TRAFs orthologues

**Figure 5:**
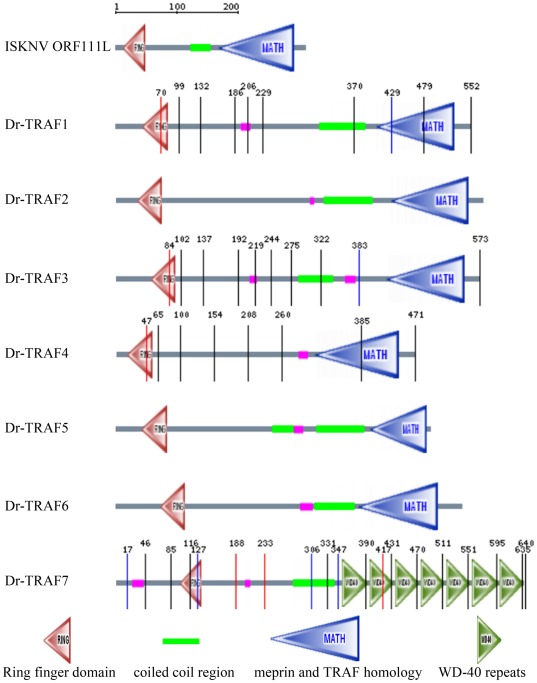
Box diagram of ISKNV ORF111L and zebrafish TRAFs. The domain architecture of ISKNV ORF111L and zebrafish TRAFs were shown using the SMART program. ISKNV ORF111L showed similar domain architecture to zebrafish TRAF1–6.

**Figure 6:**
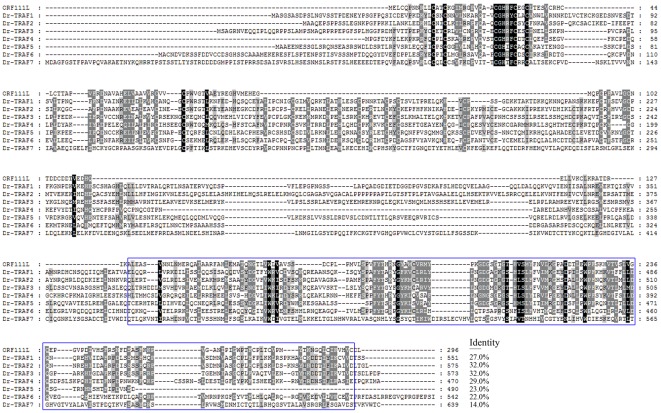
Multiple sequence alignment of ISKNV ORF111L and zebrafish TRAFs. Multiple sequence alignment was performed to compare the sequence identities between ISKNV ORF111L and zebrafish TRAFs. ISKNV ORF111L showed higher similarity with zebrafish TRAF2.

**Figure 7:**
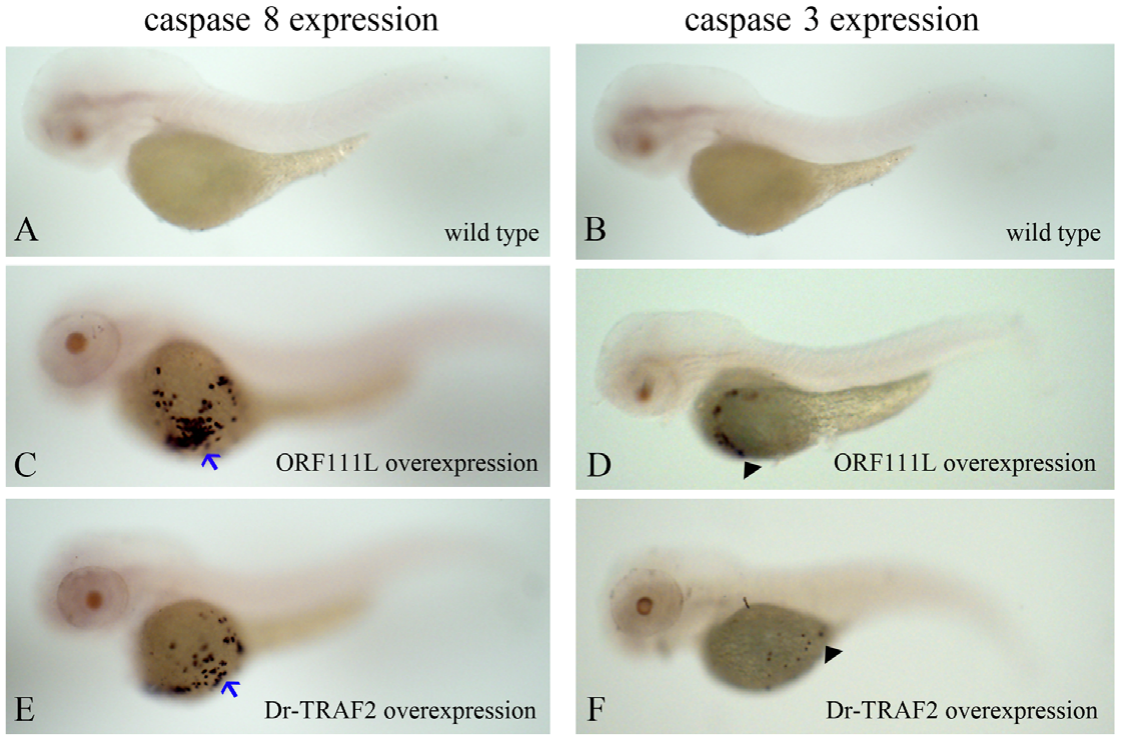
ISKNV ORF111L and zebrafish TRAF2 overexpression resulted in caspase 8 and caspase 3 upregulation. (A–B) The caspase 8 or caspase 3 expression was shown in wild type embryos by whole mount RNA in situ hybridization assay. (C–D) ISKNV ORF111L overexpression resulted in significant caspase 8 (C, arrow) and caspase 3 (D, arrow head) upregulation. (E–F) Zebrafish TRAF2 overexpression induced modest caspase 8 (E, arrow) and caspase 3 (F, arrow head) upregulation compared with those of ORF111L. All embryos shown represent the typical staining and are lateral views with anterior to the left at 3 dpf.

Bioinformatics analysis and overexpression assay were performed to show how ISKNV ORF111L (vTRAF) activated apoptosis in contrast to its cellular TRAFs counterparts. First, ISKNV ORF111L shares a similar domain architecture with zebrafish TRAF1, 2, 3, 4, 5, and 6 (Fig. 5). Second, ISKNV ORF111L had higher identities with zebrafish TRAF2 (Fig. 6) by multiple sequence alignment analysis. Third, both ISKNV ORF111L and zebrafish TRAF2 overexpression in embryo resulted in caspase 8 (Fig. 7C and 7E) and caspase 3 (Fig. 7D and 7F) upregulation by whole mount RNA in situ hybridization assay. It indicated that ISKNV ORF111L is partially similar to its cellular counterpart TRAF2. Interestingly, ISKNV ORF111L overexpression stimulated much evident caspase 8 (Fig. 7C, arrow) and caspase 3 (Fig. 7D, arrow head) upregulation than that of TRAF2 (Fig. 7E and 7F), suggesting that ISKNV ORF111L had some distinct characteristics which should be further investigated.

## Discussion

ISKNV infection induces evident apoptosis *in vitro* and *in vivo* [14]. However, the mechanism is still unknown. In the current work, the first evidence that ISKNV ORF111L, as a novel virus-encoded TRAF, interacts with TRADD and induces caspase 8-mediated apoptosis in the zebrafish model is provided. These observations may provide novel insights into the role of ORF111L in the apoptosis signalling pathway and the pathogenesis of ISKNV infection.

First, ISKNV ORF111L was demonstrated to function as a novel TRAF, which was first identified from the virus. Only four virus-encoded TRAF-like proteins were found using protein sequence comparison analyses through a searching of the NCBI and Ensembl databases, namely, ISKNV, TRBIV, RSIV, and OSGIV putative TRAF homologues. ISKNV, TRBIV, RSIV, and OSGIV are megalocytiviruses, which infect a wide range of marine and freshwater fish species [44]. The ISKNV, TRBIV, RSIV, and OSGIV putative TRAF homologues share a central TRAF domain and have high similarity with the TRAF isoforms found in fishes and mammals. Then we compared this viral TRAF with its cellular counterparts TRAFs by bioinformatics and overexpression assay. ISKNV ORF111L showed high similarity with zebrafish TRAF2, and their overexpression in zebrafish embryo resulted in caspase 8 and caspase 3 upregulation. Notablely, the upregulation effect of caspase 8 and caspase 3 by ISKNV ORF111L overexpression are much significant than that of zebrafish TRAF2. There might be two possibilities that ISKNV ORF111L induced a stronger apoptotic effect compared with its cellular counterparts. On one hand, ISKNV ORF111L might be a more direct activator of apoptosis independent of upstream pro-apoptotic signals (TRADD can interact with TNFR1 and the FAS-receptor). On the other hand, ISKNV ORF111L might be involved in or inhibit the anti-apoptotic signal pathway at the same time, which would enhance its pro-apoptotic effect.

Second, the current data reveals the mechanism of the pro-apoptotic effect of ISKNV ORF111L. Results showed that ISKNV ORF111L directly interacted with TRADD as indicated by the GST pull-down assay (Fig. 1E). In addition, ISKNV ORF111L overexpression in zebrafish embryo resulted in significant apoptosis in zebrafish embryos. In the control vector-injected embryos, the mosaic expression of GFP and RFP were observed (Fig. 2A and 3A). Most of the ISKNV ORF111L fusion proteins were mostly expressed in the yolk sac region by an unknown mechanism. In addition, we are still trying to establish the ISKNV-infected zebrafish embryo model to investigate its pathogenesis, so it is still unknown whether yolk sac is more affected by apoptosis through virus infection. Moreover, ISKNV ORF111L-induced apoptosis is associated with significant caspase 8 upregulation and activation. The caspase 8 upregulation and activation by ISKNV ORF111L overexpression became much more significant on 2 dpf and became less impressive at the later time points, suggesting that the caspase 8 activation is an early event (data not shown) as previous described [29], [45]. When the caspase 8 expression was knocked down in zebrafish by anti-sense morpholino oligos, the ISKNV ORF111L-induced apoptotic phenotype disappears, which clearly indicates that ISKNV ORF111L induces caspase 8- mediated apoptosis.

Third, the present study indicates that zebrafish is a perfect animal model for investigating some key ISKNV genes. Zebrafish has a number of advantages compared over other animal models, including rapid embryonic development and the ability to examine and manipulate embryos externally. Zebrafish has been used to study the virus–host interaction for several years, including ISKNV [44], [46], infectious hematopoietic necrosis virus (IHNV) [47], [48], [49], walleye dermal sarcoma virus (WDSV) [50], [51], hepatitis B virus (HBV) [52], spring viremia of carp virus (SVCV) [53], herpes simplex virus type 1 (HSV-1) [54], [55], tiger frog virus (TFV) [56], nervous necrosis virus (NNV) [57], viral haemorrhagic septicemia virus (VHSV) [58], spring virema of carp virus (SVCV) [59], adeno-associated virus (AAV) [60] and infectious pancreatic necrosis virus (IPNV) [61]. Adult zebrafish has been used as a ISKNV infection model, and is a valuable tool for studying the interactions between ISKNV and its host [44]. Moreover, ISKNV ORF48R functions as a new viral vascular endothelial growth factor (VEGF) using the zebrafish embryo platform [18]. A viral gene from ISKNV, which may function as a dominant-negative inhibitor of integrin-linked kinase, is currently being investigated (our unpublished data). The viral genes from ISKNV or from other virus involved in vasculogenesis, angiogenesis, cell cycle, cell fate (survival, necrosis or apoptosis), and immunology may also be investigated using the zebrafish model.

Finally, the current data verifies the potential mechanism between apoptosis and ISKNV infection. The apoptosis of infected cells is crucial for antiviral defence. One the other hand, apoptosis also facilitates the spread of viruses [62], [63], [64], [65], [66]. A previous study revealed that some viruses induce apoptosis during late stages of infection [67], and some suppresses virus-induced cellular apoptosis to facilitate viral growth during the early stages of infection [68]. Although apoptosis occurs during late stages of infection, it may facilitate the spread of infection by releasing the viral progeny to neighbouring cells while also evading host immune responses to protect the progeny viruses from host enzymes and antibodies [2], [69]. ISKNV infection induces significant apoptosis in mandarin fish fry cells [14] and adult zebrafish [14] through a poorly understood process. In the current study, we provide the first evidence that ISKNV ORF111L induces caspase 8-mediated apoptosis in the zebrafish model, which may be of great important for studies on the pathogenesis of *megalocytiviruses*. The potential therapeutic targets for ISKNV prevention are currently being investigated, and the role of ISKNV ORF111L in viral infections should be further studied.
